# Communication and action predictability: two complementary strategies for successful cooperation

**DOI:** 10.1098/rsos.220577

**Published:** 2022-09-28

**Authors:** Mateusz Woźniak, Guenther Knoblich

**Affiliations:** ^1^ Social Mind and Body Group, Department of Cognitive Science, Central European University, Vienna, Austria; ^2^ Cognition and Philosophy Lab, Department of Philosophy, Monash University, Melbourne, Australia

**Keywords:** communication, predictability, cooperation, decision making, coordination, competition

## Abstract

Making one's actions predictable and communicating what one intends to do are two strategies to achieve interpersonal coordination. It is less clear whether these two strategies are mutually exclusive or whether they can be used in parallel. Here, we asked how the availability of communication channels affects the use of strategy to make one's actions predictable. In three experiments, we investigated how people reach joint decisions if they are not allowed to communicate at all (Experiment 1), allowed minimal reciprocal communication (Experiment 2), or allowed to use the full range of conventional communication (Experiment 3). We found that when participants were not allowed to communicate, coordination was achieved by increasing action predictability. When conventional communication was allowed, there were no attempts to increase action predictability. In the minimal reciprocal communication condition, successful pairs both increased action predictability and established a communication system. Overall, this study demonstrates that people are able to flexibly adapt to coordination challenges during joint decision making and that communication reduces behavioural constraints on joint action coordination.

## 1. Introduction

The ability to cooperate and coordinate is the basis for not only being able to achieve common goals in pairs and small groups, but also laying the foundations of large societies [1–3]. It also underpins the extraordinary evolutionary success of the human race. Successful cooperation is not necessarily easy and straightforward. It requires coordination of decisions and actions between two or more people, often under uncertain and difficult circumstances. What are the cognitive mechanisms that allow humans to overcome these obstacles and make successful cooperation possible?

Schelling [4] initiated this discussion in the 1960s by proposing one strategy: doing what is predictable for others. He argued that if people need to coordinate their decisions but cannot communicate with each other, they can increase the chance of successful coordination by making their decisions predictable. A classic example is a situation where two individuals agreed to meet at a certain time but have not decided on the place of the meeting. They leave their houses and go into the city. They will succeed if they both head towards the same location (regardless of where it is), but they will lose time and effort if they head to two different locations and consequently fail to meet. Under such circumstances, people often choose a salient well-known landmark, such as Karl's Church in Vienna, or a similar outstanding spot. This strategy is known as using ‘focal points’, i.e. choosing salient options that we believe might be chosen by default by other people [5,6]. A focal point might be also a place where the involved individuals have met repeatedly before—in this case, it would be established by a history of repeated predictable behaviours [7]. These salient options come as a consequence of recursive inference made by the agents on the basis of presumed mutual knowledge.

Similar strategies exploiting other forms of predictability can be used in other situations [8,9] reflecting a general rule: coordination can be facilitated by strategic use of predictability of others' behaviour. Moreover, recent research provides rich evidence that predictability is used to facilitate cooperation and coordination across a wide range of contexts and situations going beyond decision making. When performing joint actions, people reduce the variability of their movements to smooth coordination with each other [10–13]. For example, a recent study [14] found that timing of motor action was much more predictable in a cooperative context than in a competitive one. These studies show that making one's actions more predictable is one of the basic mechanisms facilitating cooperation between individuals.

The situation when two people need to blindly meet at the same spot might seem abstract in the contemporary world in which two thirds of the world's population possess cell phones that allow anyone to simply call and discuss where to meet. Cell phones are a recent invention, but throughout the history of mankind, people have used many ways to communicate with each other to achieve common goals. Communication represents the second strategy supporting interpersonal coordination [15]. Communication is the main facilitator to achieve successful cooperation, when people can use a conventional language. However, communication can also be used to foster cooperation and coordination when a shared conventional language is not available. In such cases, people communicate through the use of sensorimotor communicative behaviours [16–18] or develop new communication systems from scratch [19–24].

It is well established that both action predictability and communication can serve as strategies facilitating successful cooperation between individuals. However, what is less explored is whether both strategies are used in an exclusive manner, or whether they can be used simultaneously, and if so, how flexible such simultaneous use is. There is evidence showing that people adapt the way that they use language to make interaction smoother [25–34], showing that predictability can be used to fine-tune communication. However, an empirical investigation of how the availability of communication modulates motivation to make one's behaviour predictable is missing. In the current study, we conducted three experiments to systematically vary the degree to which participants could communicate with each other while participating in a coordination game.

## 2. Experiment 1

The goal of the first experiment was to investigate whether people spontaneously use behavioural predictability to boost coordination with another person during a multi-trial joint decision-making task. Pairs of participants played a cooperative version of the rock–paper–scissors (RPS) game. They were instructed to cooperate to choose the same object (rock, paper or scissors). There was no possibility to communicate verbally or non-verbally. The only information that was available to participants was whether they had successfully made a joint decision or not. To increase coordination difficulty, in each trial one of the choices was randomly made unavailable. The unavailable choice was independently randomly selected for each participant. This made it impossible for the participants to coordinate by reliably choosing the same option. The reason for introducing this change was to avoid the possibility of ceiling-level performance in the cooperative task.

To establish a baseline for coordination success, the same pairs of participants also performed a modified competitive version of the standard RPS game where one participant was instructed to choose the same option as the other person, while the other participant was instructed to choose the ‘winning’ option from RPS. In all other respects, the baseline condition was the same as the cooperative condition.

We hypothesized that in the absence of any possibility to communicate, participants would make their behaviour more predictable in the cooperative condition than in the baseline condition in which participants were competing against each other. We also expected that the increased use of a strategy to make one's own choices predictable would lead participants to achieve better (and above-chance-level) performance compared with the baseline.

### 2.1. Methods

#### 2.1.1. Participants

We set our target sample size to 20 dyads (40 participants) based on power analysis indicating that such a sample will allow us to detect mid- to large-size effects (G*Power 3: *α* = 0.05, *β* = 0.8, expected d*z* = 0.45). Forty-two volunteers participated in the study comprising 21 dyads. One dyad was excluded from the analysis because one of the participants reported during the post-experiment briefing that he misunderstood the task instructions. The mean age of the remaining 40 participants was 27.45 years (s.d. = 5.1). Eighteen participants were females. There were 14 mixed-gender pairs, 2 female-only pairs and 4 male-only pairs. All participants were right-handed, except for two left-handed and one ambidextrous. The participants represented diverse national and linguistic backgrounds. All of them were fluent in English, while none was a native English speaker. Two participants reported that they did not know the RPS game prior to the experiment. All participants gave informed consent in a written form, and the study was approved by the Ethical Research Committee of Central European University (approval no. 2014/11), in accordance with the standards from the Declaration of Helsinki (applies to all experiments).

#### 2.1.2. Procedure, stimuli and apparatus

Pairs of participants played a modified RPS game. In each trial, each participant independently chose one of three objects: rock, paper or scissors. Before the experiment started, participants practised the competitive RPS game to familiarize them with the setup. During practice, participants were allowed to ask questions until they fully understood the task and setup. After the practice ended, participants were no longer allowed to verbally communicate, until the end of the experiment. After practice, participants performed a cooperative version and a baseline (competitive) version of the task (the order of versions was counterbalanced across pairs of participants)—instructions about the rules of the cooperative task were provided only immediately before the cooperative task started.

In the cooperative task, participants were instructed to make the same choice as their partner in each trial. For every trial in which both participants made the same choice (e.g. both chose paper), they both received 2 points, and for every trial in which they made different choices, they both received 0 points. In the baseline version of the task, they were playing a modified RPS game. Participant A's task was the same as in the cooperative task: to make the same choice as participant B (receiving 2 points if successful and otherwise 0 points). Participant B was instructed to choose a winning option against participant A according to the standard rules of the RPS game, i.e. rock beats scissors, paper beats rock and scissors beat paper. Participant B received 2 points for the winning option and 0 points for the non-winning option. There were three possible outcomes of each round in the baseline task: participant A wins (when both made the same choice), participant B wins (when B made a winning choice over A) and both lose (when B makes a ‘losing’ choice against A). It is important to emphasize that the individual goal of participant A was exactly the same in the cooperative task as in the baseline task: to make the same choice as participant B. Only the context of the choice differed for participant A: it was to compete in the baseline task, and to cooperate in the cooperative task.

**Figure 1.**
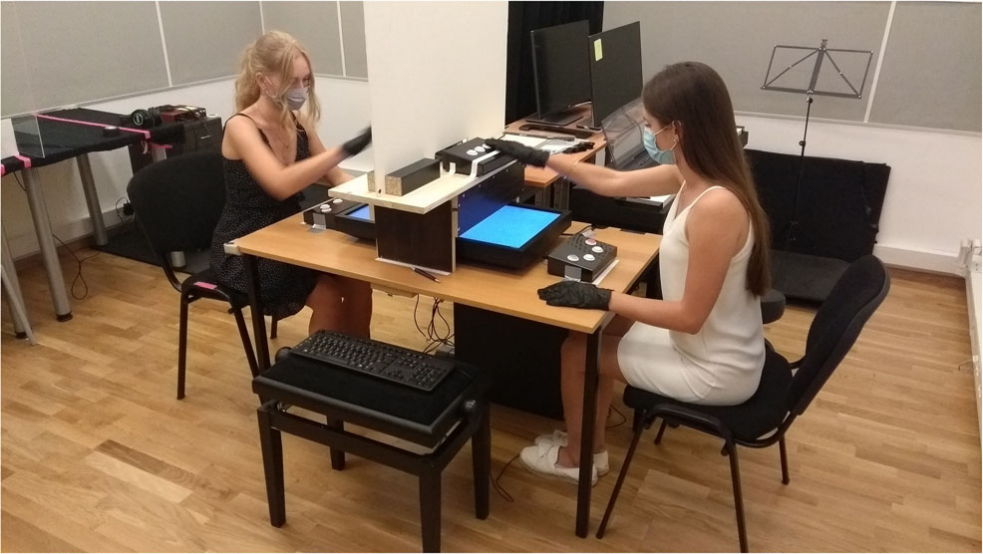
The experimental setup.

Figure 1 illustrates the experimental setup. At the beginning of the experiment, participants were seated at two sides of a table. A computer screen was placed in the lying position on the surface of a table, and a wooden structure was positioned at the centre of the table. The wooden structure served two functions. First, it prevented the participants from seeing each other and their partner's side of the screen. Second, there were two button boxes placed on top of the wooden construction, which participants used to indicate their choices; hence, they were called the ‘response boxes’. Moreover, additional button boxes were placed right in front of each participant. They were used for the participants to proceed to the next trial after giving a response, and they were called the ‘auxiliary boxes’.

**Figure 2.**
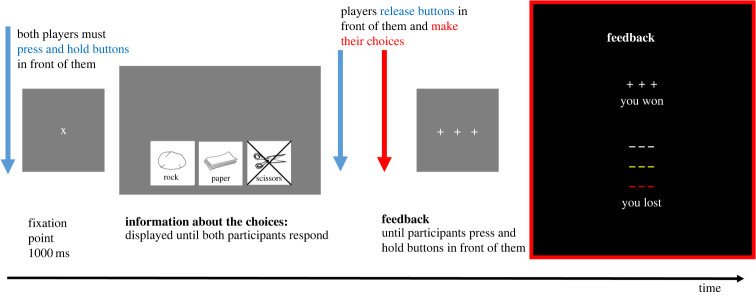
An example of the time course of a single trial for an individual participant and symbols used to indicate different types of feedback.

Figure 2 illustrates the time course of an individual trial for a single participant. Each trial started after both participants pressed and held ‘start-trial’ buttons (pink buttons in figure 1) from their auxiliary boxes. Each participant saw a fixation cross on the screen for 1000 ms. Afterwards, they saw three symbols: one on the left, one in the centre and one on the right. These symbols provided information about which button of the response box represented which choice. For example, if the symbol on the right represented a rock, then pressing the response button on the right indicated the ‘rock’ choice in a given trial. After seeing the symbols, participants decided which option to choose and pressed a button representing their choice. Importantly, in each trial one of the options was ‘blocked’. This was indicated by an ‘X’ drawn through one of the options. Participants were discouraged from choosing the blocked option because it automatically resulted in 0 points for both participants in a given trial.

The assignment of choices to the buttons of a response box (six possible combinations), as well as the blocked options, changed randomly for each participants from trial to trial—each option was blocked an equal amount of times in each experimental block. Thus, in most cases the assignment of choices to buttons and which option was blocked was different for each participant of a dyad. Importantly, the information provided about available choices for participant A was unrelated to the available choices for participant B and vice versa.

Participants were encouraged to try to make their choices at the same time as their partner. If their key presses did not occur within a 1000 ms time window, neither participant received points for the trial. The reason for this requirement was to force participants to coordinate the timing of their responses and analyse whether this was modulated by the competitive/cooperative context (these additional analyses are reported in electronic supplementary material, S1). After both participants had made their choices by pressing the relevant buttons on the response boxes, each received feedback displayed on the screen: ‘+++’ in white indicated success, ‘−−−’ in white indicated failure, ‘−−−’ in red indicated that at least one of the participants made a mistake by either choosing a blocked option or by pressing a decision button more than once, and ‘−−−’ in yellow indicated that the asynchrony between their choices was greater than 1000 ms. Feedback was presented on the screen until both participants pressed and held their fingers on the ‘start-trial’ buttons of the auxiliary boxes. This initiated the next trial.

Each dyad performed the cooperative task and the baseline task. The order of tasks was counterbalanced across dyads. For each task, participants performed 12 blocks of 18 trials. The whole experiment lasted between 40 and 60 min. The experiment was programmed and run in Matlab R2017a with Psychophysics Toolbox (v.3.0.10). All text was presented in white on a grey background.

#### 2.1.3. Research design and data analysis

The experiment comprised a single-factor within-subject design comparing a Task: cooperative versus competitive. Moreover, the competitive condition included an additional nested factor of Role (participant A versus participant B: between-subject factor). Participant A's task was to always choose the same option as participant B, while participant B had to choose the same option as participant A in the cooperative task but a winning option against participant A in the competitive task, making the latter task a version of the ‘matching pennies’ game (i.e. a game in which two participants need to make a binary choice; for example, a side of a penny coin—one participant wins if their choices are the same and the other participant wins if the choices are different).

The dependent variables were (i) win rate: average percentage of wins in a block or a task, with chance level of 33% (in both tasks for each choice made by a participant, only one choice made by the partner led to the participant's success, while two other choices by the partner led to a loss), and (ii) choice entropy: entropy of choices made by a participant within a block (measured in bits) and calculated as

2.1 $$\mathrm{Choice}\;\mathrm{entropy}=-\overset{3}{\underset{i=1}{\sum }}(\;p_{i}\log_{2}p_{i}),$$

with *p_i_* being the probability that a certain symbol *i* (rock, paper, scissors) was chosen by the participant in each block. Choice entropy reflects the predictability of choices made by a participant. For the present task, the maximum value of 1.585 bits reflects a situation in which a participant equally often chose rock, paper and scissors—meaning that the choices were random (chance level) and there was no preference for any choice over the others. A theoretical value of 0 bits would mean that a participant chose the same option (e.g. paper) in all trials of a given block, corresponding to perfect predictability. However, because in this task each option was blocked in 33% of the trials, the minimum value of choice entropy that participants could achieve was 0.918 bits, reflecting the most predictable pattern of responses (choosing one option 66% of the time and a selected second option 33% of the time).

Data analysis was conducted using custom scripts written in Matlab R2017a and using JASP 0.9.0.1. Violations of the assumption of sphericity were estimated using Mauchly's *W* test and the Greenhouse–Geisser correction was applied whenever this assumption was violated. For each frequentist analysis, we also conducted an equivalent Bayesian analysis.

All data are available at the OSF depository under the following link: https://osf.io/3eqaj/.

### 2.2. Results

In the cooperative task, 2.2% (s.d. = 2.3%) of trials were excluded because participants had made a mistake (pressing the button for the blocked option, pressing the same button twice, etc.). This amounted on average to 4.75 mistakes per participant per task (range between 0 and 19 mistakes). In the competitive task, 2.2% (s.d. = 2.3%) of trials were also excluded due to mistakes (the number of mistakes per participant also ranged between 0 and 19).

In the cooperative task, we conducted a one-way ANOVA to test whether participants were more likely to choose some options over others and we discovered that participants displayed a strong preference (*F*_2,78_ = 8.02, *p* < 0.001, partial *η*^2^ = 0.17): rock was chosen most frequently (42.2%), while paper (28.7%) and scissors (28.2%) were chosen less often. In the competitive task, we conducted a two-way ANOVA with factors of Choice (rock versus paper versus scissors) and Role (participant A versus participant B). Participants showed unequal preference for different choices as indicated by the main effect of Choice (*F*_2,76_ = 7.18, *p* = 0.001, partial *η*^2^ = 0.16): on average, they were choosing rock more often (36.7%) than scissors (32.3%) and scissors more often than paper (30.0%). Neither Role (*F*_1,38_ = 0.01, *p* = 0.91) nor Role × Choice interaction (*F*_2,76_ = 0.11, *p* = 0.90) were significant. Additional results in terms of reaction times and movement asynchronies for all experiments are reported in electronic supplementary material, S1. Additional results of analyses involving the effect of Task Order are presented in electronic supplementary material, S2.

#### 2.2.1. Win rate

A repeated-measures *t*-test revealed a significant effect of Task (*t*_39_ = 6.09, *p* < 0.001, Cohen's *d* = 0.96, BF_10_ = 41 914), showing that participants obtained significantly more points in the cooperative task than in the competitive task. A comparison of the win rate of participants A and B in the competitive task revealed no significant differences (*t*_39_ = 0.76, *p* = 0.46, Cohen's *d* = 0.24, BF_10_ = 2.58).

**Figure 3.**
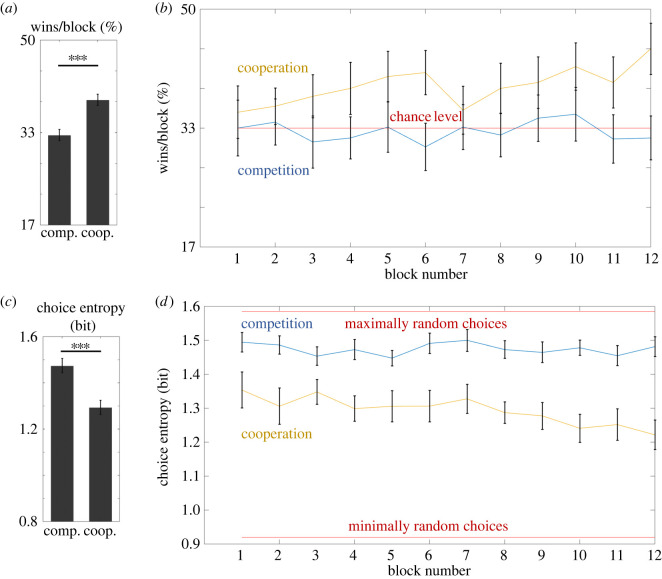
Results of Experiment 1. Bar graphs on the left illustrate the differences between competitive and cooperative tasks in terms of average win rate (*a*) and choice entropy (*c*). Line graphs on the right show average win rate (*b*) and choice entropy (*d*) across individual blocks in competitive and cooperative tasks. Brackets indicate 95% CIs.

In order to look at how coordination in the cooperative task evolved across time, in the second step we investigated the number of wins at the level of individual blocks with an additional two-way repeated-measures ANOVA (2 × 12) with factors of Task (competitive versus cooperative) and Block (from block 1 to block 12). The results revealed a significant main effect of Task (*F*_1,39_ = 37.16, *p* < 0.001, partial *η*^2^ = 0.49, BF_inclusion_ = 3.6 × 10^13^), whereas the main effect of Block was not significant (*F*_11,429_ = 1.25, *p* = 0.27 Greenhouse–Geisser corrected, partial *η*^2^ = 0.03, BF_inclusion_ = 0.002). The interaction effect was not significant (*F*_11,429_ = 1.61, *p* = 0.14, partial *η*^2^ = 0.04, BF_inclusion_ = 5.3 × 10^−4^) (figure 3).

#### 2.2.2. Choice entropy

A repeated-measures *t*-test revealed a significant effect of Task (*t*_39_ = 5.68, *p* < 0.001, Cohen's *d* = 0.90, BF_10_ = 41 914), indicating that participants displayed lower choice entropy (meaning more predictable choices) in the cooperative task than in the competitive task. A comparison of the win rate of participants A and B in the competitive task revealed no significant differences (*t*_39_ = 0.70, *p* = 0.46, Cohen's *d* = 0.22, BF_01_ = 2.66).

In the next step, we investigated choice entropy at the level of individual blocks with an additional two-way repeated-measures ANOVA (2 × 12) with the factors of Task (competitive versus cooperative) and Block (from block 1 to block 12). The results revealed that the main effects of Task (*F*_1,39_ = 32.3, *p* < 0.001, partial *η*^2^ = 0.45, BF_inclusion_ = inf) and Block (*F*_11,429_ = 3.25, *p* = 0.003 Greenhouse–Geisser corrected, partial *η*^2^ = 0.08, BF_inclusion_ = 0.017) were significant. The interaction effect was not significant (*F*_11,429_ = 2.0, *p* = 0.078 Greenhouse–Geisser corrected, partial *η*^2^ = 0.05, BF_inclusion_ = 0.003).

#### 2.2.3. Correlations between variables

In order to estimate the relationship between behavioural predictability and task success, we calculated Spearman's rho (*ρ*) correlations between choice entropy and number of wins independently for the competitive and cooperative tasks. Non-parametric correlation was used to make the results of the experiments directly comparable. In both, they were negatively correlated: in the competitive task, the correlation was *ρ*(38) = −0.384 (*p* = 0.014), and in the cooperative task, it was *ρ*(38) = −0.584 (*p* < 0.001).

#### 2.2.4. Cooperation strategies

In the cooperative task, only one cooperation strategy could ensure success: participants needed to converge on choosing one specific option whenever possible, and to choose a second specific option if the first option was blocked. For example, the participants in a pair needed to always choose rock if it was not blocked, to always choose paper if rock was blocked and to never choose scissors. Out of 40 participants, 30 reported that at some point they were using a variant of this strategy, although they often failed to be consistent in choosing a second option when the first option was blocked.

Several other strategies were also reported by the participants. Six of them reported that they were always choosing an unblocked option that would win in a standard RPS game against the other unblocked option. Three participants reported that they were trying to follow the conventional sequence of gestures: first, rock, then paper, then scissors. One participant was always pressing the middle button, and if this option was blocked, then the right button. Finally, six participants reported that they did not use any strategy at all.

**Figure 4.**
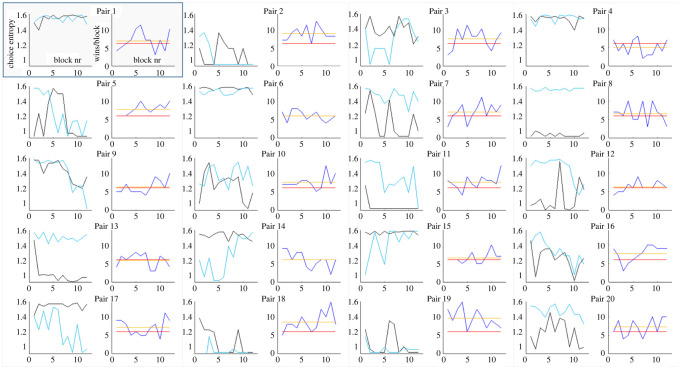
Choice entropy (graphs with black and teal lines) and numbers of wins (graphs with blue, red and orange lines) in the cooperative task across blocks for each of the 20 dyads that participated in Experiment 1. Teal lines represent participants A and black lines represent participants B. Blue lines correspond to the number of wins by each dyad in cooperative tasks in each block, while the orange lines reflect their average number of wins. The red lines correspond to the number of wins expected by chance if both participants responded randomly (six wins per block).

A complementary perspective on strategies used by participants during the cooperative task can be seen when inspecting the dynamics of choice entropy and win rate across blocks for each pair of participants (figure 4). Some pairs managed to successfully coordinate their strategies, or at least improve over time (e.g. P2, P5, P9, P11, P16, P18, P19). This was usually accompanied by an increase in win rate. In some cases, one of the participants adopted the first step of the optimal strategy (always choosing the same option, unless it was blocked), while the other did not. In such situations, the former either maintained high predictability across most of the experiment (P7, P8, P13), or became discouraged and reverted to random choice (P3, P14, P15). In both situations, the pair's win rate was usually close to chance-level performance. Finally, many pairs showed complex dynamics of choice entropy and win rate, most likely reflecting the process of unsuccessful adjustments of participants to each other.

### 2.3. Discussion

The first experiment demonstrated that individual participants were behaving in a more predictable way in the cooperative task than in the competitive task, as indicated by the significant difference in choice entropy. This difference in action predictability enabled pairs to achieve a higher win rate in the cooperative context. Most participants (75%) increased their predictability by consistently choosing the same option if it was available, which is one important ingredient of a strategy to improve joint decisions in the cooperative task. Another important ingredient is to converge on choosing not only the same option but also a second option if the preferred option is blocked. As shown by the individual results, in the absence of the possibility to communicate, only a few pairs were successful in terms of agreeing on the same prioritization of options.

We did not find any differences in the behaviour of participants A and participants B. It means that our results do not reflect the specifics of the task (whether a participant has to choose the same option as their partner or a winning option), but instead that they are fully driven by whether the context was competitive or cooperative. Moreover, the same task goal of participants A (to choose the same option as the other person) led to drastically different behaviour depending on the context of the interaction, showing that our pattern of results is not modulated by a proximal goal but reflects a general change of strategy to act in the task.

In the competitive task, entropy of choices was much higher than in the cooperative task. It was close to the theoretically possible maximum, although it did not reach that value. This finding can be explained by the fact that maximum entropy can be obtained in a situation when participants equally often choose each option within a block, i.e. six times rock, six times paper and six times scissors. This, however, would introduce a higher-order predictability into their behaviour. Therefore, the pattern of results showing choice entropy slightly lower than the theoretical maximum is what one should expect if participants tried to respond fully randomly, but it is also expected if participants engaged in some form of strategically quasi-random behaviour. This interpretation is further supported by a negative correlation between win rate and choice entropy in the competitive task.

## 3. Experiment 2

The goal of Experiment 2 was to investigate whether participants can simultaneously use two coordination strategies, predictability and communication, when both are available but neither of them alone guarantees joint success. We used the same task as in Experiment 1 but introduced a small change in the cooperative condition: each participant was allowed to send one 1-bit signal to their partner before they made their choice. We did not allow the participants to communicate in the baseline condition.

Moreover, in Experiment 2 we abandoned the distinction between participant A and participant B in the competitive context, because in Experiment 1 we did not find any evidence that this manipulation affects the results (neither directly nor through interaction). Therefore, in Experiment 2 the competitive task was the standard RPS game in which both players were guided by the same rules, to choose the winning option against their opponent.

### 3.1. Methods

#### 3.1.1. Participants

Forty volunteers comprising 20 dyads participated in the study. The mean age of the participants was 26.58 years (s.d. = 3.5). Twenty-one participants were females. There were eight mixed-gender pairs, six female-only pairs and six male-only pairs. All participants were right-handed, except for five left-handed and one ambidextrous. The participants represented diverse national and linguistic backgrounds and none of them was a native English speaker.

#### 3.1.2. Procedure

**Figure 5.**
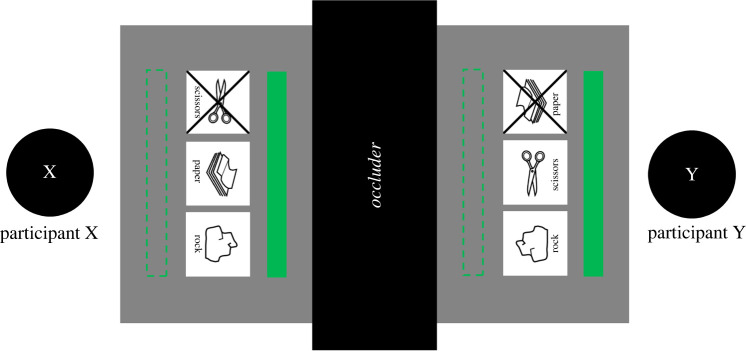
An illustration of how participants could send signals to each other in Experiment 2. In this example, participant Y sent a signal to participant X, as indicated by a solid green rectangle below the RPS pictures on Y's side of the screen, and a solid green rectangle above the RPS pictures on X's side. If participant X decides to also send a signal, then solid green rectangles will also appear in the places indicated by the dashed contours of rectangles (dashed contours were not displayed during the experiment). Each participant could send only one signal in each round and could do it only when the available RPS options were present on the screen.

The procedure of Experiment 2 was identical to Experiment 1 with two exceptions. First and most importantly, in each trial of the cooperative task participants were given a chance to send a single 1-bit signal to their partner. They could do this by pressing a ‘send-signal’ button on the auxiliary box (grey buttons in figure 1) before they made their choice using the response box. To send the signal, they used the same hand that they used to operate the other buttons. After a participant pressed a send-signal button, two green rectangles were displayed on the screen until the end of the trial—each of them visible to only one participant (see figure 5 for an example). A participant who pressed a button (sender) saw a green rectangle below the RPS images, and the other participant (receiver) saw a green rectangle above the RPS images. It was possible that both participants sent a signal, that only one of them did, or that neither of them did.

The second difference was that in the competitive context both participants had the same task, which was the standard RPS task: to choose the winning option against the other participant. If a participant chose a winning option, they received 2 points for the trial. If a participant chose a losing option or the same option, then they received 0 points.

#### 3.1.3. Research design and data analysis

The experiment comprised a single-factor repeated-measures design comparing a competitive task versus a cooperative task. Signal informativeness was added as an additional dependent variable. Signal informativeness is a measure of average informational content (in bits) conveyed by each participant within each block of the cooperative task and was calculated using the following formula:

3.1 $$\mathrm{Signal}\;\mathrm{informativeness}=-\overset{1}{\underset{i=0}{\sum }}(\;p_{i}\log_{2}p_{i}),$$

with *p*_1_ being the probability of sending a signal by a participant during a given block, and *p*_0_ reflecting the probability of not sending the signal (so, *p*_1_ + *p*_0_ = 1). Signal informativeness can take values between 0 and 1 bit, with 0 bits reflecting situations in which a participant either did not use signalling at all during a block, or was signalling in every trial (making the signal non-informative), and 1 bit reflecting a situation in which a participant was signalling during half of the trials in a given block.

Preregistration for Experiment 2 is available at: https://aspredicted.org/2j84g.pdf.

### 3.2. Results

In the competitive task, 2.2% of trials (s.d. = 1.8%) were excluded due to participants' mistakes (pressing a blocked option, pressing the same button twice, etc.). This amounted to an average 4.7 mistakes per participant (the range in both tasks was between 0 and 13 mistakes). In the cooperative task, 4.1% of trials (s.d. = 3.3%) were excluded due to participants' mistakes. This amounted to, on average, 8.95 mistakes per participant (the range in both tasks was between 1 and 29 mistakes). The difference in the number of mistakes was significantly larger for the cooperative task than the competitive task (*t*_39_ = 4.93, *p* < 0.001).

Participants did not show unequal preferences for different choices—rock, paper or scissors—neither in the competitive task (*F*_2,78_ = 0.61, *p* = 0.55, partial *η*^2^ = 0.02), nor in the cooperative task (*F*_2,78_ = 0.25, *p* = 0.78, partial *η*^2^ < 0.01).

#### 3.2.1. Win rate

A repeated-measures *t*-test revealed a significant effect of Task (*t*_39_ = 4.75, *p* < 0.001, Cohen's *d* = 0.75, BF_10_ = 783), showing that participants obtained significantly more points in the cooperative task than in the competitive task. Electronic supplementary material, S2 shows a preregistered 2 × 2 analysis, which also includes task order and additionally found that win rate in the cooperative task was higher if this task was completed first.

**Figure 6.**
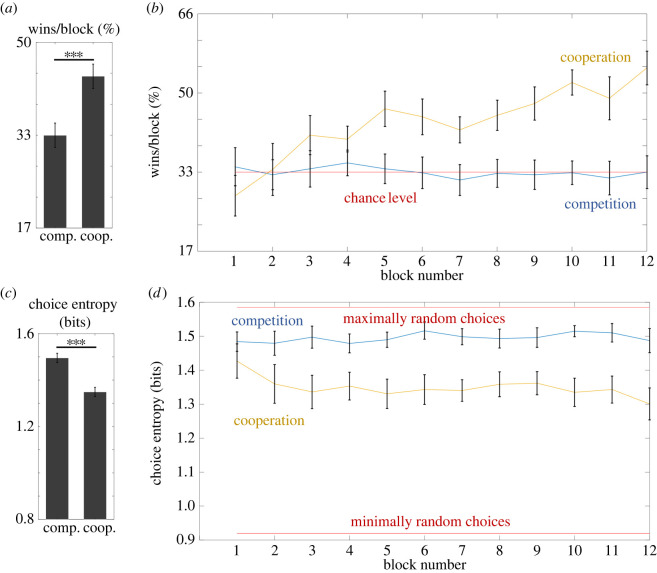
Results of Experiment 2. Bar graphs on the left illustrate the differences between competitive and cooperative tasks in terms of average win rate (*a*) and choice entropy (*c*). Line graphs on the right show average win rate (*b*) and choice entropy (*d*) across individual blocks in competitive and cooperative contexts. Brackets indicate 95% CIs.

At the second step, we investigated the number of wins at the level of individual blocks. We conducted an additional two-way repeated-measures ANOVA (2 × 12) with the factors of Task (competitive versus cooperative) and Block (from block 1 to block 12). The results revealed a significant main effect of Task (*F*_1,39_ = 22.53, *p* < 0.001, partial *η*^2^ = 0.37, BF_inclusion_ = 6.0 × 10^15^), a significant main effect of Block (*F*_11,429_ = 6.99, *p* < 0.001 Greenhouse–Geisser corrected, partial *η*^2^ = 0.15, BF_inclusion_ = 7.3 × 10^12^) and a significant interaction (*F*_11,429_ = 9.32, *p* < 0.001, partial *η*^2^ = 0.19, BF_inclusion_ = 3.7 × 10^8^) (figure 6).

#### 3.2.2. Choice entropy

A repeated-measures *t*-test revealed a significant main effect of Task (*t*_39_ = 7.09, *p* < 0.001, Cohen's *d* = 1.12, BF_10_ = 8.19 × 10^5^), indicating that participants displayed significantly lower choice entropy in the cooperative context than in the competitive context.

In the next step, we investigated choice entropy at the level of individual blocks. We conducted an additional two-way repeated-measures ANOVA (2 × 12) with the factors of Task (competitive versus cooperative) and Block (from block 1 to block 12). The results revealed that the main effect of Task (*F*_1,39_ = 50.28, *p* < 0.001, partial *η*^2^ = 0.56, BF_inclusion_ = inf) was significant. The main effect of Block (*F*_11,429_ = 1.15, *p* = 0.34 Greenhouse–Geisser corrected, partial *η*^2^ = 0.03, BF_inclusion_ = 5.4 × 10^−4^) and the interaction effect (*F*_11,429_ = 1.57, *p* = 0.15 Greenhouse–Geisser corrected, partial *η*^2^ = 0.04, BF_inclusion_ = 8.9 × 10^−5^) were not significant.

#### 3.2.3. Correlations between variables

Similarly to Experiment 1, we estimated the relationships between our dependent variables. In the competitive task, we did not find a significant correlation between choice entropy and win rate (*ρ*(38) = −0.06, *p* = 0.73), but we found a significant relationship in the cooperative task (*ρ*(38) = −0.32, *p* = 0.043) which was numerically lower than in Experiment 1. In the cooperative task, we also investigated the relationship of signal informativeness with win rate, which was strongly positive (*ρ*(38) = 0.65, *p* < 0.001). Finally and importantly, the correlation between signal informativeness and choice entropy was strongly negative (*ρ*(38) = −0.52, *p* < 0.001), meaning that participants who provided more informative signals were also making their behaviour more predictable.

#### 3.2.4. Comparison of successful and unsuccessful pairs

As the next step of the analysis, we performed an exploratory analysis in which we compared pairs that succeeded in the cooperative task with pairs that did not. To differentiate between them, we conducted *k*-means clustering (with *k* = 2) based on the average number of wins across all blocks, resulting in classification of all pairs into one of two groups. The first group contained pairs that did not succeed in the cooperative task. It consisted of 11 pairs and achieved on average 5.93 wins/block (range between 4.58 and 6.92). The second group consisted of nine pairs and achieved on average 10.31 wins/block (range between 8.83 and 13.08). The difference in the average number of wins between these groups was significant (*t*_18_ = 8.38, *p* < 0.001, Cohen's *d* = 3.77, BF_10_ = 6.9 × 10^4^).

To gain further insight into the differences between these groups, we compared them based on the dependent variables. The choice entropy of participants belonging to the successful group was only marginally smaller (*t*_38_ = 1.91, *p* = 0.064, Cohen's *d* = 0.61, BF_10_ = 1.28), but their average signal informativeness was significantly higher (*t*_38_ = 3.98, *p* < 0.001, Cohen's *d* = 1.26, BF_10_ = 84.2) compared with pairs from the unsuccessful group.

#### 3.2.5. Cooperation strategies

**Figure 7.**
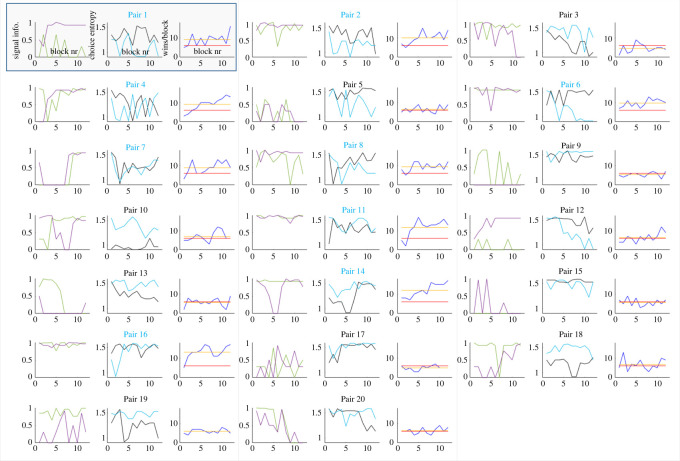
Signal informativeness (graphs with purple and green lines), choice entropy (graphs with black and teal lines) and numbers of wins (graphs with blue, red and orange lines) across blocks for each of the 20 pairs that participated in Experiment 2. In plots with signal informativeness, purple and green lines represent individual participants in each pair. In plots with choice entropy, black and teal lines represent individual participants in each pair. In plots with average wins, blue lines correspond to the number of wins by each pair in cooperative tasks in each block, while the orange lines reflect their average. The red lines correspond to the number of wins expected by chance if both participants responded randomly (six wins per block). Pairs in blue were classified as successful pairs and pairs in black as unsuccessful pairs.

In contrast to Experiment 1, in Experiment 2 there were multiple ways of succeeding in the cooperative task. Most of them required using the possibility to send signals between partners. The strategy from Experiment 1 to always choose one option whenever it was not blocked was still possible, but it was almost never successfully used, as indicated by the fact that successful pairs showed a rather high choice entropy, including in blocks in which they scored the highest points (figure 7). Instead, all successful pairs, and most pairs in general, engaged in some form of communicative behaviour. With Pair 13, one of the participants was using signalling during almost every individual trial, making the signalling uninformative. The other participant attempted to provide informative signals during the first six blocks, but afterwards stopped signalling altogether. A similar dynamic happened with Pair 15, where one of the participants was not using signalling at all, while the other started with frequent signalling but abandoned this after the first four blocks. Both pairs achieved a close-to-chance-level average number of wins.

Pairs that succeeded in coordinating their choices reported using several strategies. The simplest one included one participant sending a signal indicating a specific choice (e.g. ‘I have a rock’) and the other participant made a choice depending on this information. This one-way communication strategy did not fully use the communicative potential but was enough to achieve above-chance performance. In our case, it was used by Pair 1. All other successful pairs developed versions of a two-way communication strategy. In the most typical strategy, one of the participants was sending a signal to indicate their willingness to choose a certain option. For example, with Pair 11 one of the participants was sending a signal whenever they wanted to choose paper, while the other one was signalling whenever they wanted to choose scissors, and the pair made their decisions based on the configuration of who did and who did not send a signal. Similarly, with Pair 16 one participant was signalling paper and the other one rock; with Pair 8, it was rock and scissors; and with Pair 6, both participants were signalling if they had paper. A slightly different strategy was developed by Pair 14, where one participant was signalling whenever they had rock and the partner was either choosing rock or sending back a signal to indicate that they did not have rock. Both Pairs 7 and 4 developed a strategy in which both participants chose paper and used signalling to indicate if any of them had paper blocked. In such situations, they were choosing rock by default. Interestingly, Pair 7 started using this strategy only in the second half of the experiment, with no usage of signalling during the first half at all. Finally, Pair 2 showed a situation in which one of the partners was consistently signalling the presence of a specific option (paper), while the other person was experimenting with different communicative strategies while typically choosing scissors when in doubt.

### 3.3. Discussion

The results of Experiment 2 show that, at the group level, participants were able to use minimal reciprocal communication to improve their performance in the task. Approximately half of the tested pairs of participants developed simple communication strategies that allowed them to significantly improve their task performance. At the same time, predictability of participants' choices remained significantly higher in the cooperative context than in the competitive context, showing that participants simultaneously used both strategies—behavioural predictability and communication—to facilitate successful cooperation. Importantly, signal informativeness negatively correlated with choice entropy. It indicates that participants who were sending more informative signals were also making their behaviour more predictable, which translated to, on average, higher success in the cooperative task. Hence, successful pairs of participants used both possible strategies simultaneously to improve cooperation.

## 4. Experiment 3

Experiment 2 demonstrated that pairs of people can use both behavioural predictability and minimal forms of communication to facilitate successful cooperation. In Experiment 3, we set out to further investigate whether people may still use behavioural predictability if they are allowed to freely communicate with each other during the task. We hypothesized that, on the one hand, people might always combine communication with behavioural predictability to maximize the probability of successful coordination even if it is associated with high redundancy of coordination strategies. On the other hand, if people dynamically adjust the extent that they use communication and predictability in order to reduce the required effort, then having the possibility to freely communicate should lead to abandoning the use of predictability, as verbal communication can easily disambiguate any potential uncertainty.

### 4.1. Methods

#### 4.1.1. Participants

Forty-two volunteers participated in the study, comprising 21 dyads. One dyad was excluded from the analysis because of a malfunction of the voice-recording device. The mean age of the remaining 40 participants was 25.8 years (s.d. = 4.7). Twenty-one participants were female. There were nine mixed-gender pairs, six female-only pairs and five male-only pairs. All participants were right-handed, except for three left-handed. The participants represented diverse national and linguistic backgrounds and none of them was a native English speaker. Seven participants did not know the RPS game prior to participation in the study.

#### 4.1.2. Procedure

The procedure of Experiment 3 was identical to Experiment 2 with one exception: during the cooperative task, participants were not allowed to send signals before making their choices (just like in Experiment 1). Instead, they could speak to each other in English while the choice options were displayed on their screen (but only during this period). Their conversations were recorded and transcribed after the experiment.

#### 4.1.3. Research design and data analysis

Just as in Experiment 2, Experiment 3 comprised a single-factor repeated-measures design comparing a competitive task versus a cooperative task. Experiment 3 used the same dependent variables as Experiment 1, but also included one additional dependent variable: speech entropy.

Speech entropy is a measure of average information (measured in bits) transmitted by each participant through speech within a block of the cooperative task and was calculated using the following formula:

4.1 $$\mathrm{Speech}\;\mathrm{entropy}=-\overset{n}{\underset{i=1}{\sum }}(\;p_{i}\log_{2}p_{i}),$$

with *n* being the number of unique words used by a participant within a given block and *p_i_* being the probability that a word *i* was used by a participant within a given block (calculated as the number of times that a participant used a word *i* divided by the total number of non-unique words that a participant used during a given block). Speech entropy could take the value of 0 bits if a participant did not speak during a block. In cases when participants used spoken language during the task, speech entropy increased with the number of unique words used by a participant, but it decreased the more the distribution of usage of these words differed from a uniform distribution. For instance, if a participant used 10 unique words during a block, but 90% of the time they used only three of these words, then speech entropy would be only slightly higher than if they used only three words throughout this block (i.e. slightly higher than 1.6 bits), and much lower than if they used all 10 words equally often (i.e. 3.3 bits).

There were two reasons why we decided to use speech entropy as a quantitative measure of the amount of verbal signalling used by the participants. First, in comparison to the number of unique words, speech entropy allowed us to account for situations where only a few words were instrumental to communication, while other much less frequent words were used only as embellishments or mistakes. Second, because speech entropy was measured in bits, it became possible to compare it with choice entropy, which was also measured in bits.

Preregistration for Experiment 3 is available at: https://aspredicted.org/2k48j.pdf.

Full preregistered results for 2 × 2 ANOVAs for win rate and choice entropy are available in electronic supplementary material, S2. In the main text, we report results directly comparing competitive and cooperative tasks, as they were the focus of our main research question, which were analysed with non-parametric methods due to ceiling effects in our data in Experiment 3.

### 4.2. Results

In the competitive task, 2.2% (s.d. = 1.7%) of trials were excluded due to participants’ mistakes (pressing a blocked option, pressing the same button twice, etc.). This amounted to an average of 4.65 mistakes per participant (the range in both tasks was between 0 and 13 mistakes). In the cooperative task, 2.3% (s.d. = 3.9%) of trials were excluded due to participants' mistakes. This amounted to, on average, 5 mistakes per participant (the range in both tasks was between 0 and 39 mistakes). The number of mistakes was not significantly higher for the cooperative task than for the competitive task (*t*_39_ = −0.34, *p* = 0.73).

Participants showed a stronger preference to choose rock (37.1%) than scissors (32.2%), and scissors more than paper (29.8%) in the competitive task (*F*_2,78_ = 6.70, *p* = 0.002, partial *η*^2^ = 0.15, BF_10_ = 246). They also preferred rock (35.3%) over paper (32.7%), and paper over scissors (31.1%) in the cooperative task (*F*_2,78_ = 8.76, *p* < 0.001, partial *η*^2^ = 0.18, BF_10_ = 2444).

#### 4.2.1. Win rate

**Figure 8.**
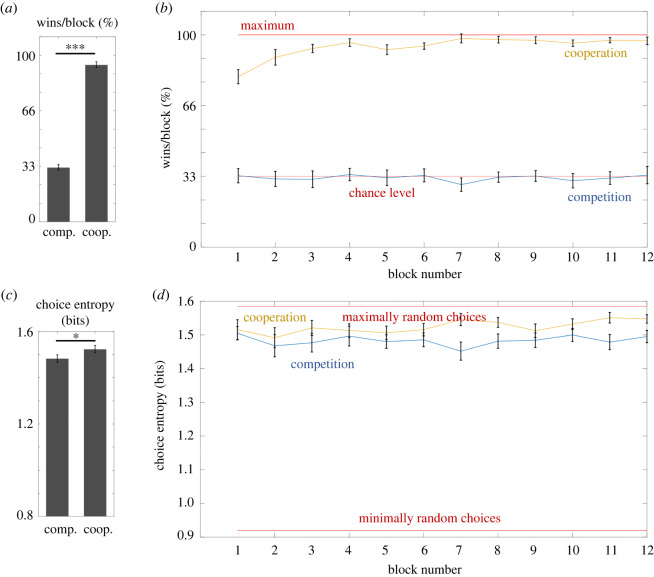
Results of Experiment 3. Bar graphs on the left illustrate the differences between competitive and cooperative tasks in terms of average win rate (*a*) and choice entropy (*c*). Line graphs on the right show average win rate (*b*) and choice entropy (*d*) across individual blocks in competitive and cooperative tasks. Brackets indicate 95% CIs.

A Wilcoxon's test revealed that participants obtained significantly more points in the cooperative task than in the competitive task (*Z*_39_ = 0, *p* < 0.001, rank biserial correlation = 0, BF_10_ = 1.49 × 10^27^). We also investigated the number of wins at the level of individual blocks with an additional two-way repeated-measures ANOVA (2 × 12) with the factors of Task (competitive versus cooperative) and Block (block 1 to block 12). The results revealed a significant main effect of Task (*F*_1,39_ = 1149.16, *p* < 0.001, partial *η*^2^ = 0.97, BF_inclusion_ = inf), a significant main effect of Block (*F*_11,429_ = 5.64, *p* < 0.001 Greenhouse–Geisser corrected, partial *η*^2^ = 0.13, BF_inclusion_ = 6.3 × 10^7^) and a significant interaction (*F*_11,429_ = 7.01, *p* < 0.001 Greenhouse–Geisser corrected, partial *η*^2^ = 0.15, BF_inclusion_ = 7.1 × 10^5^) (figure 8).

#### 4.2.2. Choice entropy

A Wilcoxon's test revealed that choice entropy was significantly higher in the cooperative task than in the competitive task (*Z*_39_ = 181, *p* = 0.002, rank biserial correlation = 0.56, BF_10_ = 2.49).

In the next step, we investigated choice entropy at the level of individual blocks. We conducted an additional two-way repeated-measures ANOVA (2 × 12) with the factors of Task (competitive versus cooperative) and Block (from block 1 to block 12). The results revealed that the main effect of Task (*F*_1,57_ = 6.12, *p* < 0.001, partial *η*^2^ = 0.14, BF_inclusion_ = 5.7 × 10^8^) was significant. The main effect of Block was not significant (*F*_11,429_ = 1.86, *p* = 0.084 Greenhouse–Geisser corrected, partial *η*^2^ = 0.05, BF_inclusion_ = 0.003), but the interaction effect was significant, although the Bayesian analysis strongly favoured its exclusion from the model (*F*_11,627_ = 2.15, *p* = 0.042 Greenhouse–Geisser corrected, partial *η*^2^ = 0.05, BF_inclusion_ = 7.6 × 10^−4^).

#### 4.2.3. Correlations between variables

As in Experiment 2 but in contrast to Experiment 1, in the competitive task we did not find a significant correlation between choice entropy and win rate (*ρ*(38) = −0.08, *p* = 0.64). In the cooperative task, this relationship was once again significant (*ρ*(38) = 0.57, *p* < 0.001). In contrast to the previous experiments, it was positive, rather than negative, meaning that better performance was associated with more random, rather than less random, choices. It is likely that pairs that needed more time to agree on linguistic conventions to use to efficiently solve the task were relying for longer on a less efficient strategy of making predictable choices. In the cooperative task, we also investigated the relationship of speech entropy with win rate (*ρ*(38) = 0.01, *p* = 0.95) and choice entropy (*ρ*(38) = 0.21, *p* = 0.19) but neither of them was significant.

#### 4.2.4. Cooperation strategies

In contrast to Experiment 2, in Experiment 3 participants were allowed to speak with each other before making a choice. This resulted in nearly perfect performance in all but one pair of participants, which however still achieved above-chance-level performance (Pair 15, average win rate across all blocks 51%). Another pair (Pair 7) performed the first half of the cooperative task in a sub-efficient manner, with only one participant communicating their choice to the partner (leading to 12.6 wins, or 70.4% win rate, in the first 6 blocks), but after a discussion during the sixth block, the pair agreed to change their strategy, leading to improvement in their performance to 99.1% win rate in the last six blocks. All remaining pairs adopted some form of a strategy that allowed near-perfect performance during the first 2–3 blocks. The most common strategy was to have one participant propose which of the three options to choose, and then have the other person approve or disapprove the choice (by saying ‘yes’/‘ok’, or ‘no’/‘blocked’). In the case of disapproval, the first person could give a second proposal, which this time was guaranteed to be correct, or in some cases the second participant could present a proposal, although with a risk that it might be blocked. This was by far the most common strategy used by 12 pairs. Three further pairs also used the strategy of proposing a common choice, but instead of using words reflecting approval or disapproval, they only used the words ‘rock’, ‘paper’ and ‘scissors’. In this case, repeating the partner's choice meant approval, while proposing a different choice meant disapproval. Two pairs used a strategy in which one person begins by saying which two options they have available (e.g. rock and paper), and then the second person makes the choice (e.g. paper). Finally, the last two pairs adopted a strategy in which the first person tells which option is blocked, and then the second person chooses which option they should select.

#### 4.2.5. Information content in verbal communication

All pairs engaged in some form of verbal communication. To estimate the quantitative characteristics and evolution across time of verbal communication between participants, we performed analyses on the transcripts of words that the participants used during the task. We calculated the number of unique words used by each participant in each block, as well as entropy of distribution of words used by each participant in each block. Speech entropy was calculated by treating speech in a similar way to choices in the RPS game: each spoken word was treated as an instance of sending one type of signal, and the distribution of the number of times that each word was used within a block served as data for calculations. Speech entropy then becomes a measure of the number of bits of information that were transmitted on average in each trial within a given block. The difference between quantifying choice entropy and speech entropy in this case is that while choices were limited to a space of only three possible options, speech was in principle unlimited.

**Figure 9.**
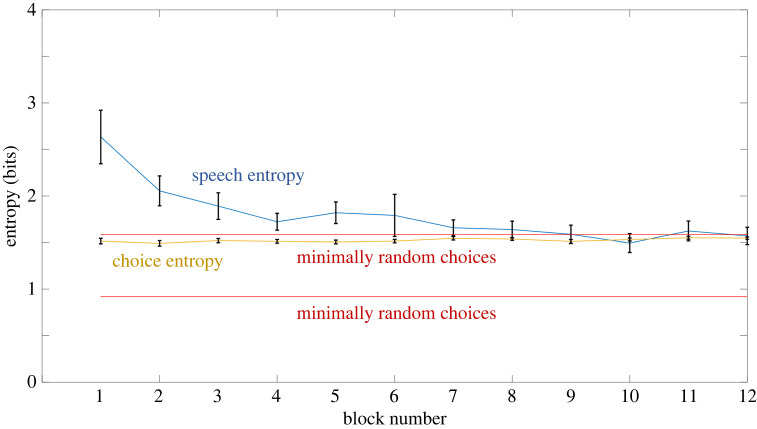
Comparison of average choice entropy and speech entropy across blocks in the cooperative task in Experiment 3. The average speech entropy was greatest in the first block and then gradually decreased until it converged with the entropy of choices, which reflected the amount of information that needed to be conveyed through verbal communication to succeed in the task, illustrating optimization of verbal communication across time. Speech entropy converged to the level of maximally random choices, because in Experiment 3 entropy of participants' choices was at that level. Brackets indicate 95% CIs.

We observed that, at the group level, both the number of unique words and speech entropy were highest in the first block, and then gradually decreased and stabilized from the seventh block onwards (figure 9). Interestingly, the average speech entropy for the whole group stabilized at the same level as choice entropy, which was also at the level of choice entropy for maximally random choices. Participants achieved this by gradually shortening and homogenizing their utterances: instead of using full sentences, they were referring only to the relevant options.

### 4.3. Discussion

Experiment 3 has shown that when unlimited verbal communication was available, all participants were able to use it to improve task performance, and all but one pair of participants used it to achieve ceiling-level performance. At the same time, the behavioural choices of participants became fully unpredictable and reached the level of choice entropy that was even higher than in the competitive context. This illustrates that behaving predictably and communicating are two interchangeable strategies of facilitating successful cooperation, and that if one of them allows the maximization of cooperation success, then there is no need to use the other anymore.

Finally, we found that the information content of verbal communication (measured by speech entropy) between participants in the cooperative context sharply decreased over time until it reached the entropy level comparable to uncertainty of participants' choices, i.e. the uncertainty that it needs to disambiguate. This shows that participants optimized their verbal communication not only to make it fully informative, but also to decrease redundancy and effort needed to coordinate.

## 5. Comparison between the experiments

As the last step, we compared the results of the three conducted experiments (preregistered analysis) for win rate and choice entropy using a 2 × 3 mixed ANOVA with factors of Task (competition versus cooperation) and Experiment (1 versus 2 versus 3). For win rate, we found a significant main effect of Task (*F*_1,117_ = 659.42, *p* < 0.001, partial *η*^2^ = 0.85, BF_inclusion_ = inf), a significant main effect of Experiment (*F*_2,117_ = 296.6, *p* < 0.001, partial *η*^2^ = 0.84, BF_inclusion_ = inf) and a significant interaction (*F*_2,117_ = 303.5, *p* < 0.001, partial *η*^2^ = 0.84, BF_inclusion_ = inf). *Post hoc* tests revealed that win rate in the competitive task did not differ between the experiments, while in the cooperative task participants were more successful in Experiment 3 than in the other experiments (both *p* < 0.001). Win rate in Experiments 1 and 2 did not significantly differ (*p* = 0.13, Bonferroni corrected).

These results show that, at the group level, participants’ win rate differed between Experiment 3 and the other two experiments. The difference between Experiments 1 and 2 was not significant, although the distribution of results in these two experiments showed different patterns. In Experiment 1, win rate was normally distributed around the mean value of 7 points (slightly above the chance level of 6 points), and the range of results was between 5 and 9.5. In Experiment 2, win rate displayed a bimodal distribution with approximately half of the pairs displaying chance-level performance, and half achieving win rates between 8 and 13 points (and sometimes approaching ceiling-level performance in individual blocks). This observation suggests that while the group-level win rate did not significantly differ between the first two experiments, the setup of Experiment 2 allowed some pairs of participants to achieve much better performance than the best pair in Experiment 1.

For choice entropy, we found a significant main effect of Task (*F*_1,117_ = 48. 2, *p* < 0.001, partial *η*^2^ = 0.29, BF_inclusion_ = 3.0 × 10^13^), a significant main effect of Experiment (*F*_2,117_ = 15.19, *p* < 0.001, partial *η*^2^ = 0.21, BF_inclusion_ = 1.0 × 10^11^) and a significant interaction (*F*_2,117_ = 24.9, *p* < 0.001, partial *η*^2^ = 0.30, BF_inclusion_ = 1.1 × 10^8^). *Post hoc* tests revealed that choice entropy in the competitive task did not differ between the experiments, while in the cooperative task participants were more successful in Experiment 3 than in the other experiments (both *p* < 0.001). Choice entropy between Experiments 1 and 2 did not significantly differ (*p* = 0.73, Bonferroni corrected). This finding shows that pairs in the first two experiments relied much more strongly on action predictability than pairs in Experiment 3.

## 6. General discussion

We conducted three experiments in which participants played a modified version of the RPS game, either cooperating with or competing against one another. Across experiments, we manipulated the degree to which participants could communicate with each other to facilitate successful cooperation. In Experiment 1, no communication was possible; in Experiment 2, participants were allowed minimal reciprocal communication; and in Experiment 3, unlimited reciprocal communication using language was allowed.

Our goal was to answer three research questions. First, we asked whether people are able to use behavioural predictability to facilitate the coordination required for joint decision making. The results of Experiment 1, in which no communication between participants was possible, provide evidence that the answer to this question is yes. Approximately half of the pairs in our task were able to achieve above-chance-level performance through making their choices more predictable in the cooperative task, making successful coordination more likely. This finding is in line with previous studies reporting similar results [4,6,12].

The second question was whether people are able to use behavioural predictability and communication, at the same time, to facilitate coordination. The results of Experiment 2 confirmed this. In a situation in which pairs of participants could use a minimal (1-bit) form of reciprocal communication, they kept ensuring behavioural predictability to succeed in the cooperative task. While not all pairs did so successfully, some achieved a level of performance that was much higher than that of the best pairs in Experiment 1. This additional benefit was due to establishing simple communicative conventions in the course of the interaction and was observed in approximately half of the pairs.

Finally, the third question was whether people still use behavioural predictability if they are allowed to use the full power of conventional verbal communication. In Experiment 3, when participants were allowed to speak with each other (unlimited reciprocal communication), we found that almost all pairs were able to rapidly reach ceiling-level performance in the cooperative task. At the same time, their choices in the task became fully random. This shows that when language was efficient enough to fully disambiguate participants' choices, participants did not need to structure their responses anymore. In fact, participants’ individual choices were less predictable when cooperating than when competing. This implies that a competitive context does not encourage fully random responses, as would be predicted by the rational agents model [35–37], but it involves a certain degree of non-randomness (for similar results see [38,39]).

When contrasting communication and behavioural predictability, our study found that the former was a much more powerful method of facilitating successful cooperation. In Experiment 1, where participants could use only behavioural predictability, they achieved performance only slightly above the chance level, while in Experiment 3, which allowed unlimited communication, all pairs except for one achieved ceiling-level performance. However, this finding should not be generalized too quickly to other joint-decision contexts. First, the design of our task made it in principle impossible to consistently achieve perfect performance in Experiment 1, whereas in Experiments 2 and 3 this option was at least theoretically within reach. Future studies could use tasks in which both strategies are equally feasible and investigate to what extent people prefer to use one over the other, and what factors might influence this preference. Second, our study involved cooperation to achieve a joint decision. In tasks that require coordination of bodily actions, such as dancing or cooperative sports, the relative influence of behavioural predictability and communication might yield an opposite pattern of results, with the former playing a much larger role.

In our study, we found that when participants could not use communication (Experiment 1), they often tried to ensure predictability of their behaviour by repeatedly choosing the same option, hoping that their partner would do the same. However, how did they decide which option to choose in the first place? One possibility is that the participants thought of one of the options as an initial focal point [4,6], i.e. an option that is more salient than the others. In the case of our task, the most salient option for participants might have been rock, as it is the first symbol that is present in the English name of the game (rock–paper–scissors) on which our study was based. Indeed, we found that participants were more likely to choose rock than the other options in the cooperative task in Experiments 1 and 3, while in Experiment 2 participants did not show preference for any option. However, in Experiment 3, participants were similarly likely to choose rock both in the cooperative context and in the competitive context, suggesting that they had an overall preference to choose this option, rather than the preference to use it in a strategic way as a focal point. In Experiment 1, participants had a significant preference to choose rock over the other options in both tasks, but this preference was stronger in the cooperative task (42.2%) than in the competitive task (36.7%). This difference provides some indication that participants might have used rock as an initial focal point. However, none of the participants mentioned that they tried to use rock more frequently in a post-experimental debriefing session. Thus, it is possible that such forms of focal points may help with coordinating decisions due to shared implicit preferences [4] (in a linguistic context see also [40]).

In Experiment 1, participants could not communicate with each other, but in Experiment 3 they could use conventional language to communicate. By contrast, Experiment 2 presented them with an intermediate situation in which they could engage in a minimal form of reciprocal communication. In order to use communication effectively, they had to develop basic communication conventions from scratch. As shown by the comparison of successful and unsuccessful pairs in Experiment 2, only about half of the tested pairs were able to bootstrap a functioning communication system. This is in line with previous studies on the development of new communicative conventions, which also found that communication success in such circumstances is highly variable [41–43]. The pairs that did succeed in our task developed various signalling conventions. In most cases, signalling was used to indicate the presence or absence of specific options—for example, sending a signal to indicate that one has or does not have scissors. Most of the successful pairs in our study developed some form of bidirectional communication, although we found pairs in which communication was predominantly unidirectional, i.e. only one of the participants was sending informative signals (Pair 1). Finally, as illustrated by the example of Pair 7, a successful communication convention can emerge spontaneously in the middle of the experiment as a novel invention, rather than in a process of gradual mutual readjustment.

We observed evolution of communicative behaviour not only in Experiment 2, where participants developed a novel (minimal) communication convention, but also in Experiment 3, where participants could use full conventional linguistic communication. In Experiment 3, we observed that the entropy of spoken communication for each participant was highest in the first block and then gradually decreased across the subsequent blocks until it reached the level of entropy representing the amount of uncertainty present in the task that participants had to disambiguate. Thus, participants were simplifying their verbal communication across the time course of the experiment until it reached the level of maximal simplicity that was needed to succeed in the task. This finding is in line with previous studies showing that, across time, people optimize their verbal communication to make it more efficient for succeeding in a joint task (e.g. [25,26,44,45]). This is most likely a specific manifestation of a more general tendency to reduce complexity or the level of required effort [46,47] that includes optimization of linguistic communication [48,49].

Our study investigated communication and action predictability treated as two separate strategies. However, on a conceptual level, they might not be fully mutually exclusive. First, acting in a predictable manner might serve a communicative function by itself. By behaving predictably, I might signal my intention to cooperate or communicate [23,50], and even communicate specific information (in the context of our task: which option I want us to converge on). At the same time, communication can be regarded as a tool for making one's behaviour more predictable. If I tell my joint action partner what I intend to do, it will make my subsequent behaviour much easier to predict. Moreover, communication can be used not only to disambiguate one's actions, but also to communicate one's level of uncertainty or predictability. This approach has been used in a study by Bahrami *et al*. [25,34] in which participants were faced with a challenging perceptual task and had to make a joint binary decision (in most of these experiments, participants could freely speak with each other). Because stimuli in this task were presented at a perceptual threshold, participants discussed not only which choice they wanted to make, but also how confident they were about what they saw. Authors of the study found that, under these circumstances, pairs of participants converged on specific vocabularies describing the confidence of their percepts and that linguistic coordination correlated with task success [25]. Interestingly, the authors also found that communication was necessary to make a group perform better than individuals, while feedback (providing knowledge about one's own and one's partner's accuracy) did not lead to such benefit. This points to a special role of communication in facilitating the success of a joint action—an effect that has been observed also in our study. However, when comparing these experiments with our study, it is important to note the differences between them. While the success of our study was defined as achieving coordination *per se*, in Bahrami *et al*. [34] the goal was to make the *correct* joint decision, not just the same decision as one's partner. This makes their task much more difficult and explains why, in our study, free communication led to ceiling-level performance, while in Bahrami *et al*. [34] it led only to a dyad-level benefit.

There are several limitations of our study. First, because our main interest was cooperation, in Experiments 2 and 3 we did not give participants the possibility to communicate in the competitive task. The reason for this was to maintain the same baseline competitive condition across all three experiments. However, future studies investigating the role of communication in a competitive context (as contrasted with the cooperative context) should implement a design in which communication is also allowed during competition. Second, we observed that in Experiment 2 the task order influenced win rate in the cooperative task. Specifically, participants were more successful if they did the cooperative task first and then the competitive task. This pattern suggests that beginning with a competitive task might, in certain cases, bias participants and make subsequent cooperation more difficult. Therefore, in future studies it might be beneficial to use a different baseline condition than competition. Finally, an open issue is to what extent our findings translate into real world situations, which are characterized by a much more complex and time-dependent structure. For example, a couple deciding how to arrange their new flat encounters a much more multidimensional coordination problem than the setup of our study. Future research should investigate to what extent the effects observed in a minimal experimental context such as ours translate to much more complex and ecologically valid situations.

## 7. Conclusion

Across three experiments we manipulated the degree to which participants could use communication to successfully achieve a joint decision. We found that behavioural predictability and communication are two complementary strategies that can facilitate interpersonal coordination and that the availability of communication reduced constraints on behavioural predictability in joint action coordination. If the bandwidth of the available communication channel is limited, people are able to simultaneously use both strategies. Even when the opportunities for communication are minimal, people are capable of bootstrapping new communication systems on the fly and to use them for cooperation.

Our study introduces a novel approach to studying cooperative social behaviour, which puts the emphasis on comparing different strategies towards achieving successful coordination. Further studies of this kind can improve our understanding of the factors that make people communicate (or not) when coordinating their actions and decisions during cooperative activities.

## Data Availability

Data are publicly available under the following link: https://osf.io/3eqaj/. The data are provided in electronic supplementary material [51].
